# Bicalutamide, an androgen receptor antagonist, effectively alleviate allergic rhinitis via suppression of PI3K–PKB activity

**DOI:** 10.1007/s00405-022-07538-w

**Published:** 2022-07-13

**Authors:** Yu Zhang, Qilei Zhang, Xinyang Wu, Geping Wu, Xingkai Ma, Lei Cheng

**Affiliations:** 1 Department of Otorhinolaryngology-Head and Neck Surgery, Lu’an Hospital of Anhui Medical University, Lu’an People’s Hospital of Anhui Province, Lu’an, 237000 Anhui China; 2 Translational Medicine Center, Zhangjiagang Hospital Affiliated to Soochow University, Suzhou, 215000 China; 3 College of Biomedical Engineering of Eye Optometry Medicine, Wenzhou Medical University, Wenzhou, 325000 China; 4 Department of Otolaryngology, Zhangjiagang Hospital Affiliated to Soochow University, Suzhou, 215600 China; 5 Department of Otorhinolaryngology & Clinical Allergy Center, the First Affiliated Hospital, Nanjing Medical University, 300 Guangzhou Road, Nanjing, 210029 China

**Keywords:** Allergic rhinitis, AR/PI3K–PKB, Bicalutamide

## Abstract

**Objectives:**

To investigate the therapeutic effect of Bicalutamide, an androgen receptor antagonist on the onset and development of allergic rhinitis in an animal model.

**Methods:**

40 male BALB/c mice were randomly divided into five groups (eight mice per group). Aluminum hydroxide powder was used as an adjuvant, combined with Ovalbumin (OVA) to establish the mouse model of allergic rhinitis via ultrasonic nebulization of OVA to stimulate the nasal cavity. Mice in Bica#1 group were intraperitoneally injected with 0.02 mg Bicalutamide/0.5 ml of normal saline daily for 7 consecutive days; mice in Bica#2 group were administered 0.02 mg Bicalutamide/0.5 ml of normal saline via intraperitoneal injection for 5 consecutive days, and then the same amount of normal saline was injected intraperitoneally for 2 consecutive days. Enzyme-linked immunosorbent assay was adopted to detect the serological levels of IgE, IL-4, and IL-6 production. Eosinophil infiltration was observed under microscope after hematoxylin and eosin staining of nasal mucosa. Quantitative PCR and Western blot were employed for determination of histamine receptors mRNA expression and PI3K/PKB associated protein levels, respectively.

**Results:**

Histological analysis shown that allergic lesion was induced after OVA sensitization. Intraperitoneal injection with 0.02 mg Bicalutamide daily for 7 consecutive days significantly reduced the allergic lesion; however, mice injected with the same amount of normal saline at the same time demonstrated no allergic rhinitis symptoms. In addition, there was a significant reduction in eosinophils number in Bicalutamide treated mice (*n* = 8) compared to the OVA group (*n* = 8) (OVA: 19.6 ± 5.3 vs. Bica#1: 7.7 ± 0.8 vs. Bica#2: 9.4 ± 1.2, both *p* < 0.01). Furthermore, ELISA results revealed that the serological levels of IgE (OVA: 17.3 ± 1.7 µg/ml vs. Bica#1: 9.2 ± 0.6 vs. Bica#2: 10.4 ± 2.3, both *p* < 0.05), IL-4 (OVA: 164.3 ± 5.1 pg/ml vs. Bica#1: 110.2 ± 3.1 vs. Bica#2: 115.3 ± 4.1, both *p* < 0.05) and IL-6 (OVA: 167.3 ± 3.7 pg/ml vs. Bica#1: 117.5 ± 6.5 vs. Bica#2: 114.8 ± 2.4, both *p* < 0.05) were significantly decreased after two different dosage of Bicalutamide treatment. Similarly, histamine receptors in mast cells were significantly reduced after two different dosage of Bicalutamide treatment. More importantly, p-PKB protein was notably reduced after two different dosage of Bicalutamide treatment compared to the OVA group, mTOR protein levels were also down regulated after two different dosage of Bicalutamide treatment.

**Conclusions:**

Our data demonstrated that androgen receptor antagonist Bicalutamide can significantly alleviate allergic rhinitis lesion in the animal model. PI3K/PKB activity in mast cells was suppressed after Bicalutamide injection. Our results provide important implication in allergic rhinitis prevention and treatment.

## Introduction

Allergic rhinitis is a common and frequently-occurring disease in clinical practice. Because of its complex pathogenesis, the clinical treatment effect is not satisfactory. Mast cells are the main receptor cells in the pathogenesis of allergic rhinitis [1]. They cause early symptoms of allergic rhinitis by secreting substances, such as histamine, bradykinin, protease and arachidonic acid derivatives, and stimulate and trigger other symptoms of allergic rhinitis [2]. Granulocytes involved in late response increased. Activated mast cells secrete numerous cytokines, both preformed and newly synthesized, that modulate T- and B-cell function, amplify early and late manifestations of inflammatory responses, and lead to changes in tissue structure [3]. Therefore, elucidating the mechanisms that regulate activation, apoptosis and their aggregation into tissues in mast cells is of great significance for understanding the pathogenesis of allergic rhinitis and providing targets for novel drug treatments. In the past decade, there has been intensive research into the regulation of multiple biological functions by signaling in mast cells, and the pathogenesis of mast cell-related diseases. Phosphatidylinositol 3-kinase/protein kinase B (PI3K–PKB), including upstream regulatory molecules, downstream targets, antagonists of various receptors, and inhibition of intracellular signaling factors, etc., these may become potential therapeutic drugs for allergic diseases [4, 5].

PI3K–PKB is a family of intracellular signaling proteins. The catalytic activity of the catalytic subunit in the PI3K holoenzyme is critical for mast cell activation, differentiation, survival and homeostasis. It also plays an important role in the acute increase in mast cell activation and vascular permeability in IgE-induced early allergic responses [6, 7]. Our previous animal studies [8] also found that large doses of α-tocopherol can inhibit the onset of allergic rhinitis through the PI3K–PKB pathway. Therefore, PI3K/PKB pathway can be potential target candidate in treatment of allergic inflammation. It further revealed that inhibition of cell migration by Akt/PKB was mediated by Glycogen Synthase Kinase-3β (GSK-3β) [9]. In the study of drug resistance in the treatment of prostate cancer, it was found that there is reciprocal feedback regulation of PI3K and androgen receptor signaling [10]. The feedback inhibition of the androgen receptor can inhibit PI3K pathway, i.e., blockade of the androgen receptor leads to down regulation of AKT phosphorylation and thus suppression of the PI3K pathway [11, 12]. Bicalutamide is a nonsteroidal androgen receptor antagonist that inhibits the androgen receptor [11]. Therefore, we speculate that Bicalutamide can alleviate the occurrence and development of allergic rhinitis via suppressing PI3K/PKB activity.

## Materials and methods

### Experimental equipments and reagents

Ultra-micro spectrophotometer (BD-1000), electronic balance scale (Mettler ME104), low-temperature high-speed benchtop centrifuge (Thermo Heraeus fresco 17), micro-sampler gun (Thermo Finnpipette), Light Cycler TM PCR (Roche), Electric thermostatic water bath (Shanghai Jinghong Experimental Equipment Co., Ltd.), microtome (Leica CM1950), IL-4, IL-6 and IgE kits (R&D Systems), ovalbumin, OVA (Sigma-Aldrich), aluminum adjuvant (Sigma-Aldrich), dexamethasone tablets (Sinepharm Pharmaceutical, Shanghai), Bicalutamide Tablets (Shanghai Zhaohui Pharmaceutical).

### Experimental animal of allergic rhinitis

Forty BALB/c mice, male, 7–8 weeks, all weighing about 20 g, were purchased from Shanghai Slack Laboratory Animal Co., Ltd. These mice were randomly divided into five groups (eight mice/group). Group A. Control group, B. Allergic rhinitis group, C. Dexamethasone group, D. Bicalutamide treatment group #1, E. Bicalutamide treatment group #2. Experimental mouse model of allergic rhinitis was established as previously reported [13], aluminum hydroxide powder was used as an adjuvant, combined with OVA to establish the mouse model of allergic rhinitis via ultrasonic nebulization of OVA to stimulate the nasal cavity. One-hundred µg of OVA and 2 mg of aluminum hydroxide powder were dissolved and evenly mixed in 1 ml of normal saline. OVA–saline suspension were injected intraperitoneally into the mice in groups B, C, D and E every other day. Group A was intraperitoneally injected with the same amount of normal saline at the same timepoint. Local challenge from the 22nd day: 20 ml of 1% OVA saline was used for ultrasonic atomization, once a day, for 15 min each time, and the treatment continued for 1 week. Mice in group A were nebulized with equal volume of normal saline at the same time. All animal experiments in this study followed the guidelines for Institutional Animal Care at Zhangjiagang Hospital affiliated to Soochow University. The animal protocol was approved (#20200717).

From the 22nd day, mice in group C were given 0.2 mg dexamethasone /0.5 ml of normal saline every day. Mice in group D were intraperitoneally injected with 0.02 mg Bicalutamide/0.5 ml of normal saline daily for 7 consecutive days; mice in group E were administered 0.02 mg Bicalutamide/0.5 ml of normal saline via intraperitoneal injection for 5 consecutive days, and then the same amount of normal saline was injected intraperitoneally for 2 consecutive days; mice in groups A and B were intraperitoneally injected with the same amount of normal saline at the same time, and the mice were observed the allergic rhinitis symptoms for 7 consecutive days.

### Collection of peripheral serum and nasal mucosa

After the last ultrasonic atomization and drug intervention at the end of the fourth week, mice in each group were anesthetized by intraperitoneal injection of 10% chloral hydrate. The nasal cavity specimens of mice were collected, placed into 10% formaldehyde solution for fixation after dissection, and then decalcified with 20% EDTA, embedded, and sliced ​​to collect nasal mucosal tissue.

### Cytokine detection

The experiment used double antibody one-step sandwich enzyme-linked immunosorbent assay (ELISA). To the coated microwells pre-coated with mouse IL-4, IL-6 and IgE antibodies, add the sample, standard, standard, and horseradish peroxidase-labeled detection antibody in sequence, and incubate and thoroughly washing. Use the substrate TMB to develop color, TMB is converted to blue under the catalysis of peroxidase, and converted to final yellow under the action of acid, and a stop solution is added. The optical density (OD) value was measured at 450 nm, the concentrations of IL-4, IL-6 and IgE were proportional to the OD value, and the standard curve was drawn to obtain the concentrations of IL-4, IL-6 and IgE in the samples.

### Staining of nasal mucosa and nasal mucosa eosinophils

Hematoxylin–Eosin (HE) staining: sections of nasal mucosa tissue were stained with HE, and the number of eosinophils in the nasal mucosa of mice in different groups was observed under a microscope.

Count the number of eosinophils in 5 fields of view under a microscope at high magnification (× 400) for each nasal mucosa specimen, and take the average value.

### Quantitative real-time PCR

Trizol LS (Invitrogen) solution was used for lysis of nasal mucosa, followed by RNAqueous™ Total RNA Isolation Kit (Thermo Fisher). The total RNA extracted was then measured by UV spectrophotometer, and D260/280 and D260/230 were calculated. Total RNA was subjected to RNA reverse transcription reaction using Promega reverse transcription kit in the following reaction system. 5xBuffer 5.0 µL, random primers 0.5 µL, total RNA 8.5 µL, 10 mM dNTP (Promega) 2.0 µL, RNase inhibitor (Promega) 0.5 µL, M-MLV (Promega) 1.0 µL to total volume to 20 µL, and after mixing, incubated at 42 °C for 60 min; then incubated at 85 °C for 10 min to inactivate reverse transcriptase. The real-time quantitative PCR reaction system is as follows: 5.0 µL of cDNA, 0.5 µL of forward primer and 0.5 µL of reverse primer, 10 µL of 2xSYBR GreenPCR MasterMix, 4.0 µL of ddH2O, and a total volume of 20 µL. The reaction conditions are as follows: 95 °C, 5 min; 95 °C for 15 S, 60 °C for 15 S, 72 °C for 30 S, a total of 40 cycles on Light Cycler TM PCR (Roche). The expression level of mRNA was calculated with 2^−ΔΔCt^.

### Detection of PI3K/PKB pathway by western blot

Take out part of the nasopharyngeal tissue from the sacrificed mice, grind it with liquid nitrogen to fully rupture the cells, put the broken tissue into a glass mixer, add an appropriate amount of lysis buffer at 1 ml/1 mg, containing PMSF, and incubate on ice 30 min, then centrifuge at 4 °C for 5 min at 12,000 r/min, suck the supernatant, put it in a − 80 °C refrigerator for freezing and save for later use. Total tissue protein was quantified by Coomassie brilliant blue. The tissue protein samples were mixed with 1/4 ratio the amount of 5xSDS loading buffer, boiled and denatured for about 6 min, 10% SDS–PAGE 115 V for electrophoresis, 210 mA constant current for 60 min, and wet transferred to nitrocellulose membrane (NC membrane, Invitrogen). After blocking with 5% nonfat milk powder in TBST buffer for 2 h at room temperature, the membranes were treated with anti-PKB (Cell signaling technology CST#9272), p-PKB monoclonal antibody(CST#4060), GSK3β(CST#9832), mTOR antibody (CST#2983) (1:500). The membrane was washed 3 times with TBST, 10 min each time, and then incubated with goat anti-rabbit or mouse IgG conjugated with horseradish peroxidase (1:5000) at room temperature for 1 h. The membrane was washed with TBS 3 times, 10 min each time, and finally the luminescence kit ECL was used. The reaction was developed by exposure to X-film.

### Statistical analysis

SPSS 22.0 software was used for data statistics, all data are expressed as Mean ± Standard deviation (*X* ± s). One-way ANOVA was used to compare data among multiple groups. When there was statistical significance, that is, *p* < 0.05, pair-wise comparison was performed and least significant difference (LSD) method was exceeded by the difference between two varietal means for a particular characteristic. Error bar values represent SEM. For comparison between two groups, Student’s two-tailed t test was used.

## Results

### Bicalutamide inhibits allergic rhinitis in mouse model

**Fig. 1:**
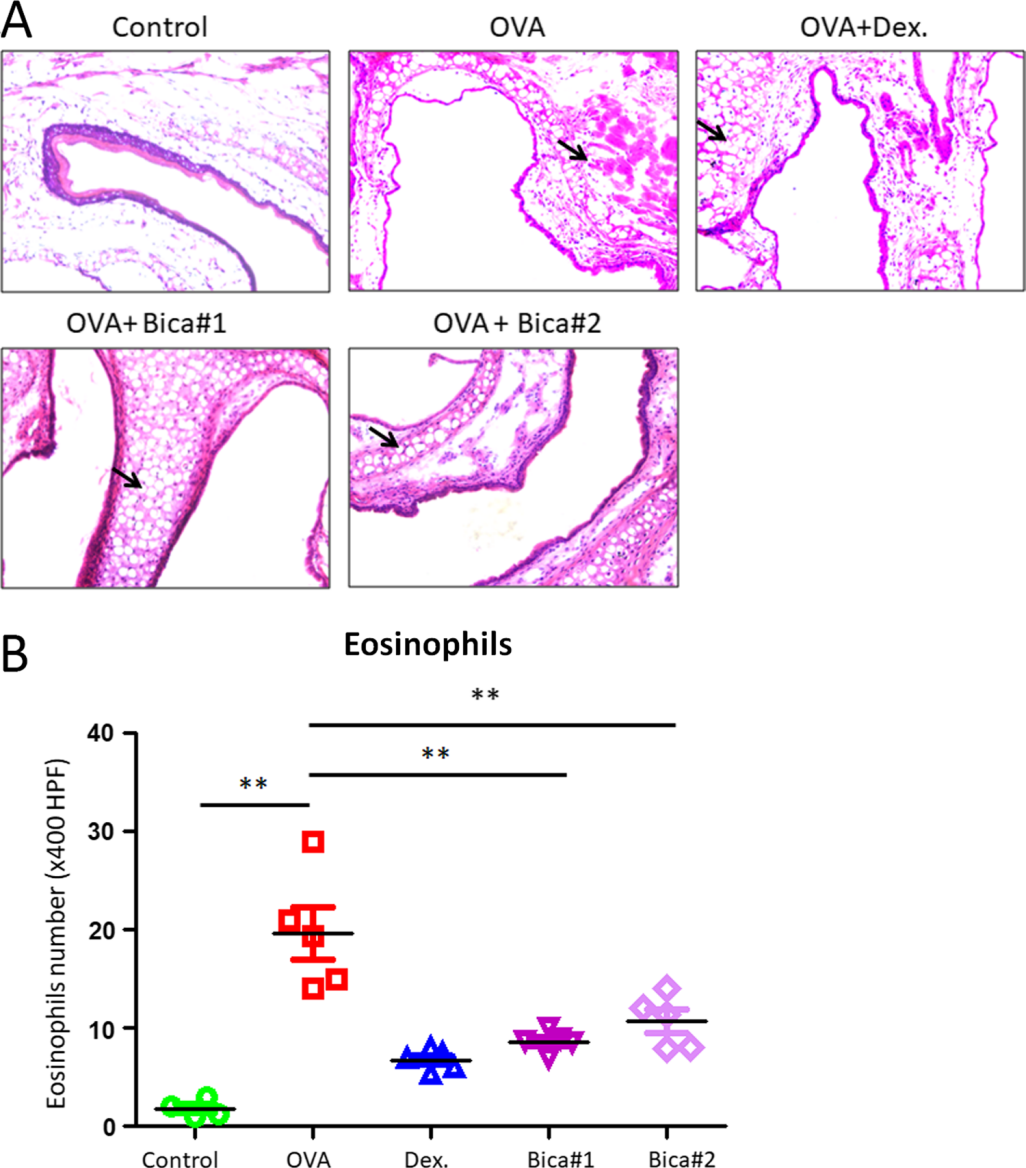
Bicalutamide inhibits allergic rhinitis in mouse model. **A** Representative sections of the nasal mucosa tissue under hematoxylin and eosin staining microscope (× 400). *n* = 8 each group. Magnification: × 400. Data are representative of at least two independent experiments. **B** Number of eosinophils in the nasal septal mucosa. *n* = 8 each group. Data are representative of at least two independent experiments. **p < 0.01. Error bar values represent SEM. For comparison between two groups, Student’s two-tailed t test was used

Histological analysis shown that allergic lesion was induced after OVA sensitization (Fig. 1A). Intraperitoneal injection with 0.02 mg Bicalutamide daily for 7 consecutive days significantly reduced the lesion of allergic rhitinis (Fig. 1A); Another dosage of i.p. administration with 0.02 mg Bicalutamide for 5 consecutive days, and then the same amount of normal saline was injected intraperitoneally for 2 consecutive days, showing the allergic lesion was alleviated compared to OVA group (Fig. 1A). There was a notable reduction in inflammatory cells infiltration in cross sections in Bicalutamide treated mice compared to the OVA group. However, mice injected with the same amount of normal saline at the same time demonstrated no allergic rhinitis symptoms. Eosinophil counts in the nasal mucosa are shown in Fig. 1B. There was a significant reduction in eosinophils in Bicalutamide treated mice (*n* = 8) compared to the OVA group (*n* = 8) (OVA: 19.6 ± 5.3 vs. Bica#1: 7.7 ± 0.8 vs. Bica#2: 9.4 ± 1.2, both *p* < 0.01). The eosinophil number was significantly lower in Dex group compared to OVA group; there were no significant changes of eosinophils between Dex group and Bica-1,-2 groups.

### Bicalutamide inhibits serological levels of IgE, IL-4 and IL-6

**Fig. 2:**
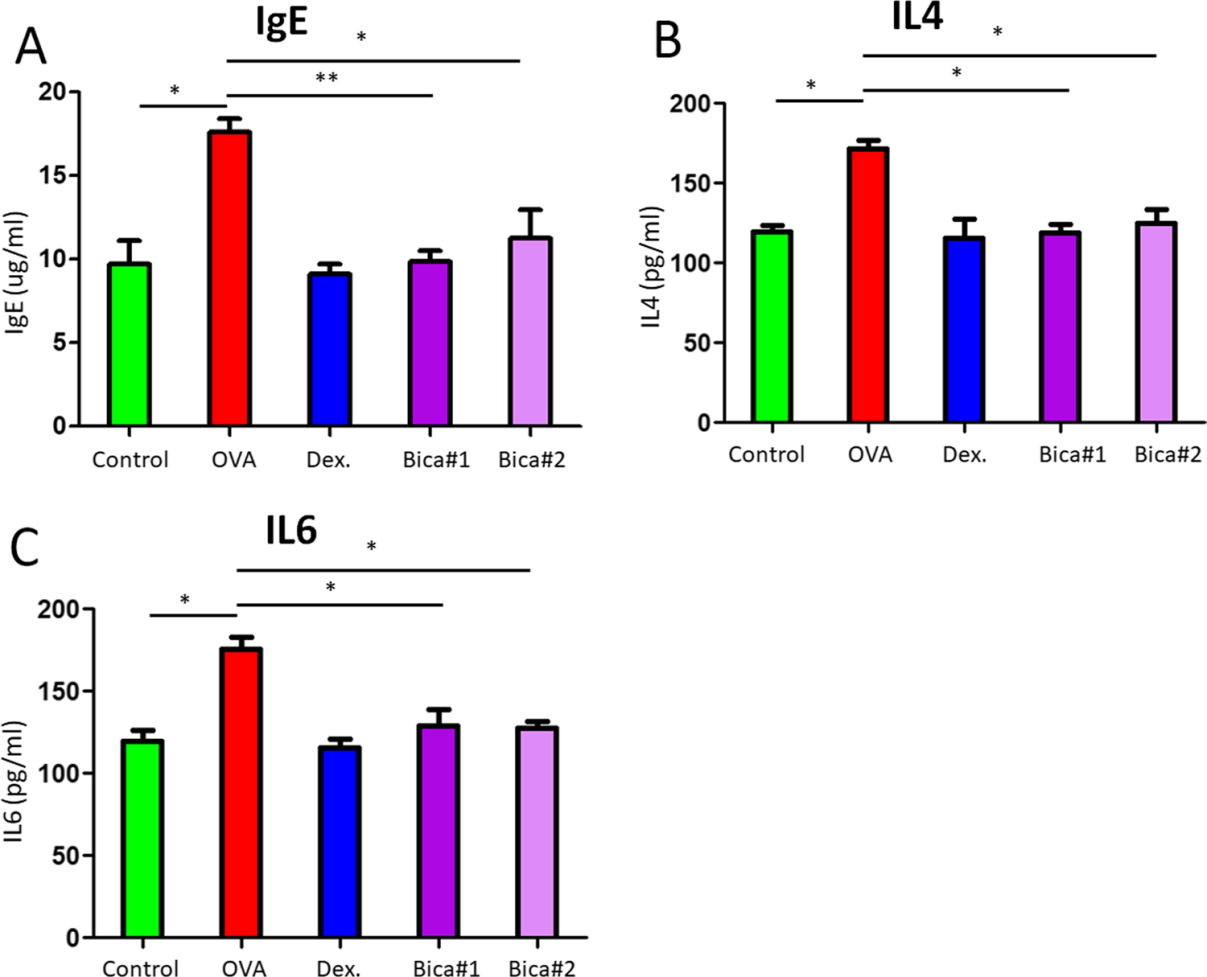
Bicalutamide inhibits serological levels of IgE, IL-4 and IL-6. Comparison of serological levels of IgE, IL-4, and IL-6 production between groups. **A** IgE; **B** IL-4; **C** IL-6. *p < 0.05; **p < 0.01; Error bar values represent SEM. For comparison between two groups, Student’s two-tailed t test was used

There was a significant increase in IgE, IL-4, and IL-6 production in serum collected from OVA group versus that from control group (Fig. 2). However, different dosage treatment of Bicalutamide significantly suppressed the levels of IgE (OVA: 17.3 ± 1.7 µg/ml vs. Bica#1: 9.2 ± 0.6 vs. Bica#2: 10.4 ± 2.3, both *p* < 0.05), IL-4 (OVA: 164.3 ± 5.1 pg/ml vs. Bica#1: 110.2 ± 3.1 vs. Bica#2: 115.3 ± 4.1, both *p* < 0.05) and IL-6 (OVA: 167.3 ± 3.7 pg/ml vs. Bica#1: 117.5 ± 6.5 vs. Bica#2: 114.8 ± 2.4, both *p* < 0.05) production in serum compared with those in the OVA group (Fig. 2A–C).

### Histamine receptors in mast cells were significantly reduced after Bicalutamide treatment

**Fig. 3:**
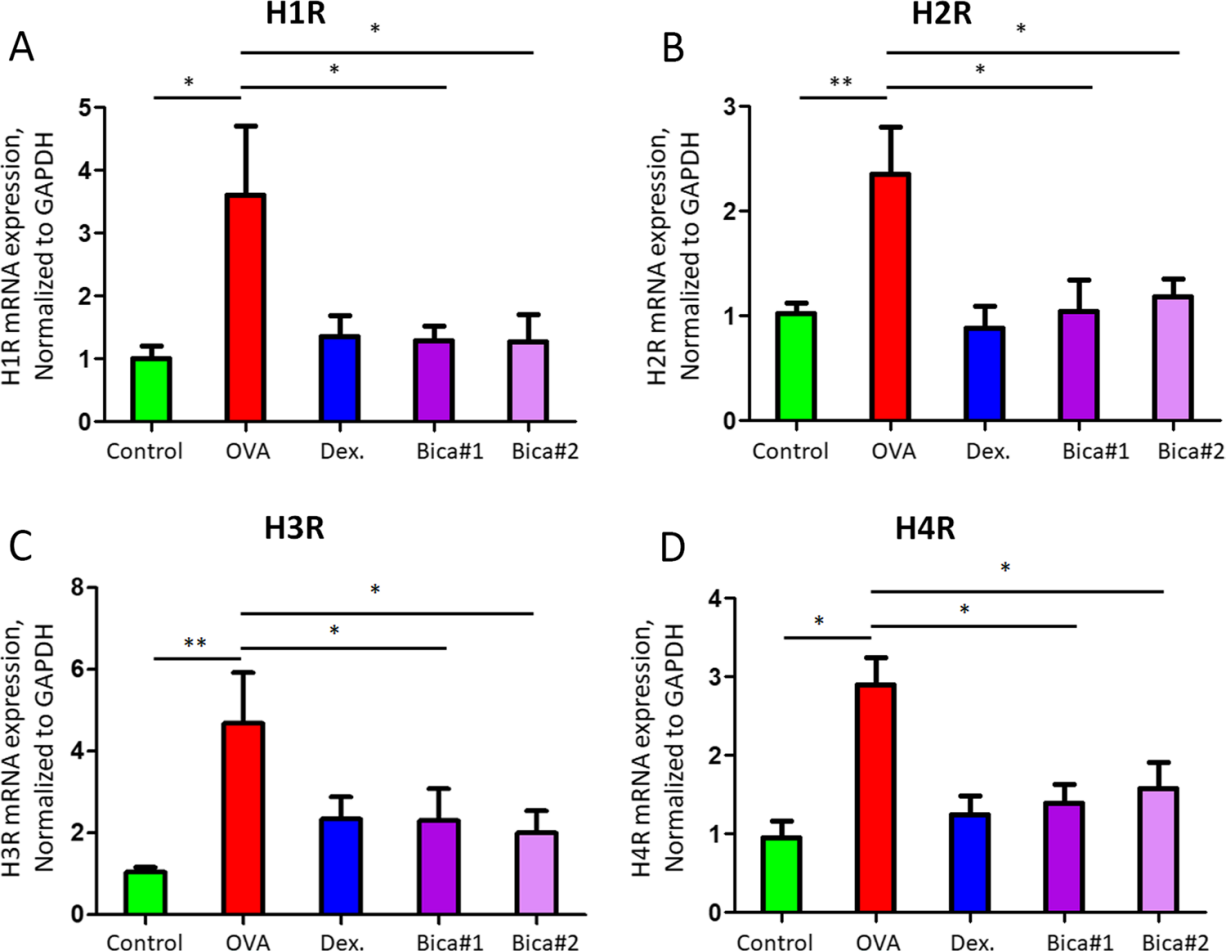
Quantitative PCR detection for histamine receptors mRNA expression. **A** H1R; **B** H2R; **C** H3R; **D** H4R. *p < 0.05; **p < 0.01; Error bar values represent SEM. For comparison between two groups, Student’s two-tailed t test was used

Histamine and its receptors (H1R–H4R) play a crucial and significant role in the development of various allergic diseases [2]. We further examined the histamine receptors expression in nasal mucosa tissues. Single-cell suspensions of the nasal mucosa tissues (1 × 10^6^) collected from OVA and Bicalutamide treated mice were lysed for total RNA extraction and quantitative PCR detection for histamine receptors mRNA expression (n = 8 for each group); the results demonstrated that mRNA levels of histamine receptors including H1R, H2R, H3R, and H4R was significantly reduced after different dosage treatment with Bicalutamide compared to those in OVA group (Fig. 3A–D).

### Bicalutamide suppressed PI3K–PKB activity

**Fig. 4:**
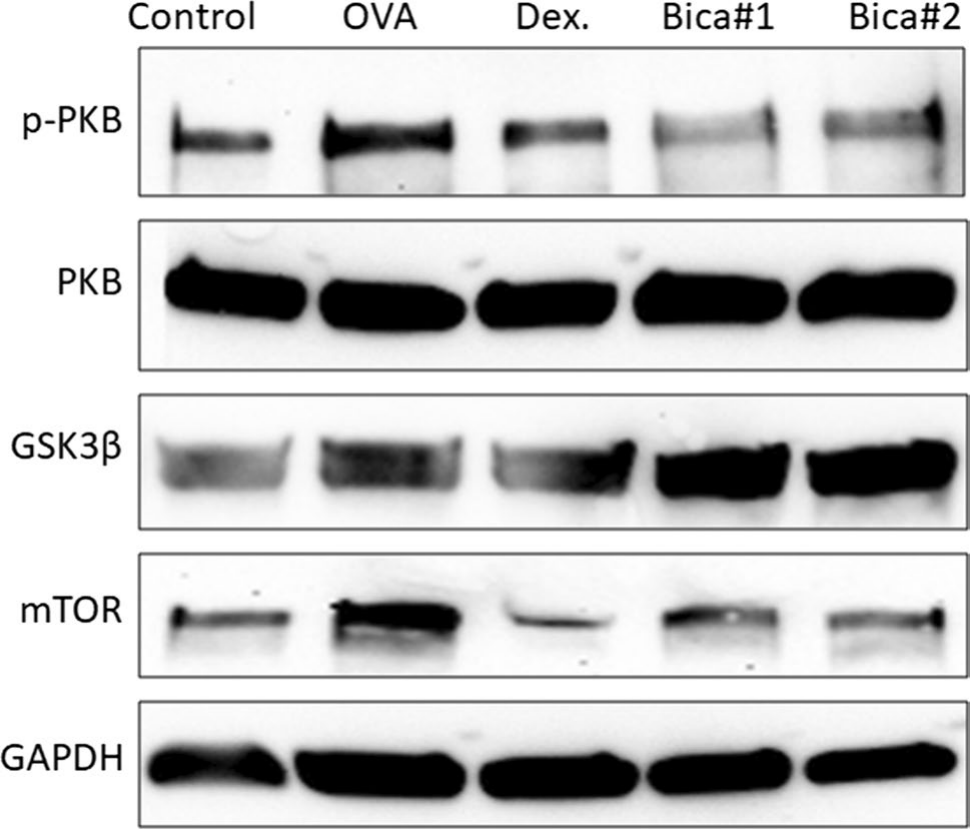
Western-blot to detect protein levels of p-PKB, PKB, GSK3b and mTOR. GAPDH protein was selected as loading control

We further examined the protein level of PKB expression and PKB targets GSK3β and mTOR expression. Western-blot demonstrated that p-PKB protein was reduced after Bicalutamide treated mice compared to the OVA group (Fig. 4), further mTOR protein levels were also down regulated after Bicalutamide treatment. However, GSK3β was increased in both dosage treatment with Bicalutamide compared to OVA group. PKB total protein was not significantly changed between groups (Fig. 4).

## Discussion

The immune response of allergic rhinitis originates from secondary exposure to allergens, and the symptoms are divided into two stages: early response and late response. Regardless of the early or late stage, the response of effector cells such as mast cells and eosinophils and their products are the key factors in the entire immune response [14]. In this study, OVA and aluminum hydroxide were used as sensitizers to replicate the mouse model of allergic rhinitis by intraperitoneal injection and ultrasonic nebulization, which is a relatively mature and reliable method. Histological analysis in our study shown that allergic lesion was induced after OVA sensitization (Fig. 1A). Intraperitoneal injection with 0.02 mg Bicalutamide daily for 7 consecutive days significantly reduced the lesion of allergic rhitinis (Fig. 1A); Another dosage of i.p. administration with 0.02 mg Bicalutamide for 5 consecutive days, and then the same amount of normal saline was injected intraperitoneally for 2 consecutive days, showing the allergic lesion was alleviated compared to OVA group (Fig. 1A). These data demonstrated that Bicalutamide treatment is similar to Dexamethasone effect in allergic rhinitis alleviation in animal model.

Allergic rhinitis is an allergic inflammation mainly infiltrated by immune cells including eosinophils. Results in our study demonstrated that there was a notable reduction in inflammatory cells infiltration in cross sections in Bicalutamide treated mice compared to the OVA group. However, mice injected with the same amount of normal saline at the same time demonstrated no allergic rhinitis symptoms. Eosinophil counts in the nasal mucosa are shown in Fig. 1B. There was a significant reduction in eosinophils in Bicalutamide treated mice compared to the OVA group. Allergen-specific T helper 2 (Th2) cells and elevated serum IgE are typical manifestations of allergic rhinitis, and the significantly elevated levels of IL-4 and IL-6 also indicate that two cytokines play an important role in the disease process of allergic rhinitis [14]. The pathology of allergic rhinitis shows the accumulation and increase of mast cells, dendritic cells, macrophages, etc. in the superficial nasal mucosa, as well as the levels of inflammatory factors such as IL-4, IL-6, IL-8, and IgE were increased. Allergens can induce a Th2-related immune response in a subset of CD4 + T cells, secrete inflammatory factors, such as IL-4, IL-5, and IL-13, and mediate the occurrence of humoral immunity [15]. There is evidence that T cell-mediated Th2 cytokines including IL-4, IL-5, and IL-13 are elevated in patients with allergic rhinitis, and can regulate eosinophil growth and differentiation [16]. Results in our study shown there was a significant increase in IgE, IL-4, and IL-6 production in serum collected from OVA group versus that from control group (Fig. 2). However, different dosage treatment of Bicalutamide significantly suppressed the levels of IgE, IL-4, and IL-6 production in serum compared to those in the OVA group (Fig. 2A–C). One of the most well-known mast cell activation pathways is the high-affinity IgE receptor (FcεRI), which degranulates mast cells and releases intracellular mediators. When this process is uncontrolled and enhanced, it can lead to an allergic inflammatory response. The mucosal epithelial cells of the nasal cavity also have the function of antigen-presenting cells. The surface of these cells expresses the IgE receptors FcεRI and FcεRII, and can synthesize and release a variety of cytokines and inflammatory mediators during allergic reactions, including eosinophil chemokines, mast cell secreting cytokines, intercellular adhesion molecule 1 (ICAM1), IL-6, IL-8, Tumour necrosis factor α (TNFα) and vascular endothelial growth factor (VEGF). Therefore, treatments potentially targeting these pathways can be considered as novel methods to control the development of allergic rhinitis. Similar treatments such as dexamethasone are often used for the treatment of allergic rhinitis by intranasal, oral or intravenous routes. The application of Bicalutamide in the intervention study of allergic rhinitis has not been reported before. Therefore, the differential dosage treatment of Bicalutamide in the current study is insightful to potential clinical use in allergic rhinitis.

Activation of PI3K channels often occurs in the following situations: activation of growth factor receptors or PIK3CA for core coding of PI3KAalpha, through loss-of-function phosphatase and deletion of the tensin homolog on chromosome 10 (PTEN), through protein kinase B (PKB/Akt) up regulation, or damage to the spinal sclerosis complex TSC1/2. When PI3K is activated, such as phosphorylation or activation of AKT, it localizes to the cell membrane. Activated AKT has many functions, such as activation of CREB, inhibition of p27, localization of FOXO in the cytoplasm, activation of PtdIns-3 ps [4] and mTOR, which can affect the transcription of p70 or 4EBP1. A number of factors are now known to enhance PI3K/AKT channels, including EGF, Shh, IGF-1, insulin and calmodulin. Meanwhile, this channel is also antagonized by various factors, including PTEN [17], GSK3β and HB9 [5]. Previous animal studies [8] also found that large doses of α-tocopherol can inhibit the onset of allergic rhinitis through the PI3K–PKB pathway. In the current study, we found that p-PKB protein was reduced after Bicalutamide treatment compared to the OVA group (Fig. 4), further mTOR protein levels were also down regulated after Bicalutamide treatment. However, GSK3β was increased in both dosage treatment with Bicalutamide compared to OVA group. PKB total protein was not significantly changed between groups (Fig. 4). PI3K pathway regulate the expression of histamine H4 receptors in mast cells and thus can regulate the release of cytokines, such as TNF-α and IL-8, while the release of TNF-α and IL-8 is inhibited by inhibitors of PI3K signaling, suggesting that H4 receptors can regulate the release of TNF-α and IL-8 in human cells through PI3K signaling [8]. In addition, in the study of prostate cancer, it was also found that the overexpression of microRNAs (miRNAs) directly responded to the overactive PI3K pathway, reduced the expression level of target genes to a certain extent, and inhibited the downstream regulators of the PI3K–PKB pathway. It can lead to increased expression of miRNA target genes [18]. GSK-3β functions in diverse cellular processes that have been implicated in a range of human pathologies, including allergic asthma [19]. GSK3β-related expansion of Treg cells was one potential mechanism that Broncho-Vaxom attenuates allergic airway inflammation in an animal model [20]. Molecularly GSK-3β has been shown to activate the downstream components of the PI3K/AKT/mTOR signalling cascade by phosphorylating AKT, RICTOR, TSC1 and 2, PTEN and IRS1 and 2 [21]. Results in our study shown increased levels of GSK3β after the treatment with Bicalutamide compared to OVA group, indicating that GSK-3β was activated under Bicalutamide treatment. Further investigation is required for exploring the immunological mechanism by Bicalutamide in treating allergic rhinitis.

The role of histamine and histamine receptors are demonstrated in mast cell-mediated allergy and inflammation [2]. As the most studied mast cell meson, histamine is formed in the Golgi by histidine decarbonation and stored in mast cell granules and basophils. When mast cells are exposed to a specific antigen, histamine is released in a calcium-dependent degranulation manner. Histamine levels in nasal secretions increased in a bimodal pattern lasting approximately 1 min immediately after nasal allergen challenge and between 3 and 11 h. These responses occur in its recipient cells and peripheral blood leukocytes, including eosinophils, neutrophils, CD4 + T cells, basophils and mast cells. It is now believed that there are three classical histamine receptor subtypes (H1, H2, H3) and a new subtype, H4. These histamine-induced allergic disease symptoms are mainly caused by the interaction between H1 receptors. H2 receptors are involved in the production of gastric acid and to some extent in the symptomatic response of the respiratory tract. H3 receptors have been implicated in controlling both histamine production and release from nerve tissue. Although the clinical role of the H4 receptor has not been established, it appears to overlap in structure and function with the H3 receptor. Recent studies have shown that the expression of histamine H4 receptors in mast cells can regulate the release of proinflammatory cytokines and chemokines, such as the colony cell stimulating factor TNF-α and the interleukin IL-8. The release of TNF-α and IL-8 was inhibited by inhibitors of PI3K, ERK and Ca2 + -Calcineurin–NFAT signaling pathway, while the release of pro-inflammatory factors and chemokines was inhibited by P38 signaling pathway. Furthermore, activation of H4R triggers the phosphorylation of ERK, p38 and AKT in mast cells. Results in our current study demonstrated that the mRNA levels of histamine receptors including H1R, H2R, H3R, and H4R were significantly reduced after different dosage treatment with Bicalutamide compared to those in OVA group (Fig. 3A–D). Further study is required to explore the mechanism that Bicalutamide inhibited histamine receptors, thus could regulate the release of TNF-α and IL-8 in mast cells through PI3K, Ca2 + –Calcineurin–NFAT and MAPKs signaling pathways.

## Conclusions

Bicalutamide, a non-steroidal androgen receptor antagonist, demonstrated a therapeutic role in allergic rhinitis in an animal model, further our results indicated activation of PI3K–PKB cascade was significantly blocked by Bicalutamide, which can shown as a new direction on clinical drug prevention and treatment of allergic rhinitis. Some adverse effects by Bicalutamide, including hot flashes, breast swelling, nausea, vomiting, and trouble sleeping, need to be considered before clinic use in allergic rhinitis.

## References

[1] Galli SJ, Grimbaldeston M, Tsai M (2008). Immunomodulatory mast cells: negative, as well as positive, regulators of immunity. Nat Rev Immunol.

[2] Thangam EB, Jemima EA, Singh H, Baig MS, Khan M, Mathias CB, Church MK, Saluja R (2018). The role of histamine and histamine receptors in mast cell-mediated allergy and inflammation: the hunt for new therapeutic targets. Front Immunol.

[3] Modena BD, Dazy K, White AA (2016). Emerging concepts: mast cell involvement in allergic diseases. Transl Res.

[4] King D, Yeomanson D, Bryant HE (2015). PI3King the lock: targeting the PI3K/Akt/mTOR pathway as a novel therapeutic strategy in neuroblastoma. J Pediatr Hematol Oncol.

[5] Ojeda L, Gao J, Hooten KG, Wang E, Thonhoff JR, Dunn TJ, Gao T, Wu P (2011). Critical role of PI3K/Akt/GSK3beta in motoneuron specification from human neural stem cells in response to FGF2 and EGF. PLoS ONE.

[6] Ali K, Bilancio A, Thomas M, Pearce W, Gilfillan AM, Tkaczyk C, Kuehn N, Gray A, Giddings J, Peskett E, Fox R, Bruce I, Walker C (2004). Essential role for the p110delta phosphoinositide 3-kinase in the allergic response. Nature.

[7] Horak F, Puri KD, Steiner BH, Holes L, Xing G, Zieglmayer P, Zieglmayer R, Lemell P, Yu A (2016). Randomized phase 1 study of the phosphatidylinositol 3-kinase delta inhibitor idelalisib in patients with allergic rhinitis. J Allergy Clin Immunol.

[8] Wu G, Zhu H, Wu X, Liu L, Ma X, Yuan Y, Fu X, Zhang L, Lv Y, Li D, Liu J, Lu J, Yu Y (2020). Anti-allergic function of alpha-Tocopherol is mediated by suppression of PI3K-PKB activity in mast cells in mouse model of allergic rhinitis. Allergol Immunopathol (Madr).

[9] Yoeli-Lerner M, Chin YR, Hansen CK, Toker A (2009). Akt/protein kinase b and glycogen synthase kinase-3beta signaling pathway regulates cell migration through the NFAT1 transcription factor. Mol Cancer Res.

[10] Crumbaker M, Khoja L, Joshua AM (2017). AR signaling and the PI3K pathway in prostate cancer. Cancers (Basel)..

[11] Carver BS, Chapinski C, Wongvipat J, Hieronymus H, Chen Y, Chandarlapaty S, Arora VK, Le C, Koutcher J, Scher H, Scardino PT, Rosen N, Sawyers CL (2011). Reciprocal feedback regulation of PI3K and androgen receptor signaling in PTEN-deficient prostate cancer. Cancer Cell.

[12] Massard C, Chi KN, Castellano D, de Bono J, Gravis G, Dirix L, Machiels JP, Mita A, Mellado B, Turri S, Maier J, Csonka D, Chakravartty A (2017). Phase Ib dose-finding study of abiraterone acetate plus buparlisib (BKM120) or dactolisib (BEZ235) in patients with castration-resistant prostate cancer. Eur J Cancer.

[13] Fan EZ, Xi L, Han DM, Zhang SZ, Li Y, Zhang L (2009). Evaluation of the safety of aluminium adjuvant in the preparation of allergic rhinitis animal model. Zhonghua Er Bi Yan Hou Tou Jing Wai Ke Za Zhi.

[14] Cheng L, Chen J, Fu Q, He S, Li H, Liu Z, Tan G, Tao Z, Wang D, Wen W, Xu R, Xu Y, Yang Q (2018). Chinese Society of allergy guidelines for diagnosis and treatment of allergic rhinitis. Allergy Asthma Immunol Res.

[15] Bui TT, Kwon DA, Choi DW, Jung SY, Lee SY, Piao CH, Hyeon E, Fan Y, Yeon SH, Son RH, Shon DH, Song CH, Shin HS (2019). Rosae multiflorae fructus extract and its four active components alleviate ovalbumin-induced allergic inflammatory responses via regulation of Th1/Th2 imbalance in BALB/c rhinitis mice. Phytomedicine.

[16] Fan Y, Piao CH, Hyeon E, Jung SY, Eom JE, Shin HS, Song CH, Chai OH (2019). Gallic acid alleviates nasal inflammation via activation of Th1 and inhibition of Th2 and Th17 in a mouse model of allergic rhinitis. Int Immunopharmacol.

[17] Wyatt LA, Filbin MT, Keirstead HS (2014). PTEN inhibition enhances neurite outgrowth in human embryonic stem cell-derived neuronal progenitor cells. J Comp Neurol.

[18] Dart DA, Uysal-Onganer P, Jiang WG (2017). Prostate-specific PTen deletion in mice activates inflammatory microRNA expression pathways in the epithelium early in hyperplasia development. Oncogenesis.

[19] Wu X, Wang S, Han M, Song B, Ye P, Ma S, Li J, Chen F, Xu G, Ding Q, Xia J, Li H (2015). Critical link between glycogen synthase kinase 3beta and forkhead box P3 in patients with chronic rhinosinusitis with nasal polyps. J Allergy Clin Immunol.

[20] Fu R, Li J, Zhong H, Yu D, Zeng X, Deng M, Sun Y, Wen W, Li H (2014). Broncho-Vaxom attenuates allergic airway inflammation by restoring GSK3beta-related T regulatory cell insufficiency. PLoS ONE.

[21] Hermida MA, Dinesh Kumar J, Leslie NR (2017). GSK3 and its interactions with the PI3K/AKT/mTOR signalling network. Adv Biol Regul.

